# Supplementary material for: Rapid diagnosis of periprosthetic joint infection from synovial fluid in blood culture bottles by direct matrix-assisted laser desorption ionization time-of-flight mass spectrometry
Source: PLoS One. 2020 Sep 24;15(9):e0239290. doi: 10.1371/journal.pone.0239290 (PMC7515592; doi:10.1371/journal.pone.0239290)
Supplement: S1 File — (DOC) [file pone.0239290.s002.doc]

長庚醫學研究計畫申請書

| 一、基本資料 | | | | | | | | |
| --- | --- | --- | --- | --- | --- | --- | --- | --- |
| 計畫類別 | | | ☑個別型計畫  🞎整合型計畫 | 整合型總計畫主持人 | | |  | |
|  |  |  |  | 整合型總計畫名稱 |  | | | |
|  |  |  |  |  |  |  |  |  |
|  |  |  | ☑醫學類🞎工程類🞎管理學類🞎教學類🞎Top-down🞎目標導向🞎臨床試驗  🞎長庚醫學研究資料庫🞎研究平台維持計畫🞎資料庫維持計畫🞎科研究中心(含中心維持計畫)🞎其他 | | | | | |
| 計畫性質 | | | 🞎純基礎研究☑應用研究🞎技術發展🞎醫學教育 | | | | | |
| 主持人 | | | 姓名：郭峯志 服務單位：高雄長庚醫院骨科 | | | | | |
|  |  |  | 職稱：學術組助理教授級主治醫師 貢獻比：30% | | | | | |
| 共同主持人(二人以上，請自行插入表格) | | | 姓名：李禎祥 服務單位：高雄長庚醫院感染科 | | | | | |
|  |  |  | 職稱：學術組副教授級主治醫師 貢獻比：30% | | | | | |
| 協同主持人(二人以上，請自行插入表格) | | | 姓名：李炫昇 服務單位：高雄長庚醫院骨科 | | | | | |
|  |  |  | 職稱：學術組教授級主治醫師 貢獻比：15% | | | | | |
| 協同主持人(二人以上，請自行插入表格) | | | 姓名：王俊聞 服務單位：高雄長庚醫院骨科 | | | | | |
|  |  |  | 職稱：學術組教授級主治醫師 貢獻比：15% | | | | | |
| 協同主持人(二人以上，請自行插入表格) | | | 姓名：林柏君 服務單位：高雄長庚醫院骨科 | | | | | |
|  |  |  | 職稱：學術組助理教授級主治醫師 貢獻比：10% | | | | | |
| 計畫  名稱 | 中文 | | 使用基質輔助雷射脫附離子化質譜儀技術來快速診斷人工關節感染 | | | | | |
|  | 英文 | | Rapid diagnosis of prosthetic joint infection by matrix-assisted laser desorption ionization time-of-flight mass spectrometry | | | | | |
| 執行期限 | | | 全程計畫：自民國105年12月01日起至民國108年05月31日  本年度計畫：自民國105年12月01日起至民國106年11月30日 | | | | | |
| 研究學門/審查組別 | | | 研究學門：檢驗醫學  審查組別：微生物與免疫學(見下頁) | | | | | |
| 本年度申請主持長庚研究計畫共1件。(共同主持之計畫不予計入)  本件在本年度所申請之計畫中優先順序(不得重複)為第1。 | | | | | | | | |
| 本計畫是否為跨院校合作☑否；　¨是，合作機構：  本計畫是否為國際合作計畫☑否；　¨是，合作國家： | | | | | | | | |
| 計畫連絡人 | | 郭峯志 | | | | 院/校內分機 | | 8003 |
|  |  |  |  |  |  | 院內院內GSM後五碼 | | 56445 |

計畫中文摘要：請於五百字內就本計畫要點作一概述，並依本計畫性質自訂關鍵詞。

| **關鍵詞：** 質譜儀，診斷，人工關節感染，基質輔助雷射脫附離子化    人工關節感染是人工關節置換最嚴重的併發症之一。一旦發生人工關節感染，最重要的是要能正確及快速地找到致病菌，才能得到有效的治療。傳統細菌培養及敏感藥物測試常常會有偽陰性的結果產生，特別是當人工關節表面形成生物膜時。同時，關節液或是人工關節旁的組織檢體必須至少培養14天以上，才能有較正確的結果產生。使用血液培養瓶來診斷人工關節感染的致病菌已行之多年。它已被證實能有效改善診斷的準確性及特異性。但使用血液培養瓶仍然耗時，一般平均需21~23小時才能培養出結果。    近期基質輔助雷射脫附離子化質譜儀的出現，已經被採用當作是快速從血液培養瓶中培養出致病菌的工具。使用這個方法時，只要幾滴的血液在自陽性血液培養瓶取出執行此檢驗方法不到1個小時即可檢查出病原菌。而且基質輔助雷射脫附離子化質譜儀只需少量的細菌即可診斷。在文獻上，當使用基質輔助雷射脫附離子化質譜儀診斷尿液培養的周轉時間，已大幅從24小時降到4~6個小時即可檢查出細菌。但是否可以用基質輔助雷射脫附離子化質譜儀來快速診斷人工關節感染，目前仍是個未知數。  這個2年期的計劃，第一年要納入25個有人工關節感染的病人。第二年再納入另外25個有人工關節感染的病人。而這些有人工關節感染的病人，將持續追蹤感染後接受階段性再次置換人工關節後的結果。在人工關節感染的病人接受手術清創時，所取得檢體將以基質輔助雷射脫附離子化質譜儀進行微生物偵測。我們預期使用基質輔助雷射脫附離子化質譜儀將能大大縮短診斷細菌所需的時間。 |
| --- |

計畫英文摘要：請於五百字內就本計畫要點作一概述，並依本計畫性質自訂關鍵詞。

| **Keywords:** mass spectrometry, diagnosis, prosthetic joint infection, matrix-assisted laser desorption ionization  **Background**  Prosthetic joint infection (PJI) remains one of the most catastrophic complications after total joint replacement. To achieve the efficacy of treatment, the causing microorganism must be identified accurately and rapidly.    Conventional culture and sensitivity test on agar plates often have falsely negative results as biofilm are formed on the prosthesis. Meanwhile, the synovial fluid or periprosthetic tissue specimens must be incubated for at least 14 days. Blood culture bottles (BCBs) have been used to identify the pathogens in PJI with the improvement of sensitivity and specificity. But BCBs still take considerable time, within a median of 21 to 23 hours, to yield positive results.    Recently, matrix-assisted laser desorption ionization time-of-flight mass spectrometry (MALDI-TOF MS) has been adopted as a rapid tool to identify bacteria from positive blood culture bottles. In this method, only a few drops of blood form positive blood bottle and subjected to MALDI-TOF MS for a short time (less than one hour) with a small amount of an organism. The turnaround time for identification of urine culture was improved from 24 hours to 4-6 hours when using MSLDI-TOF MS compared with conventional cultures. But it is unknown if MSLDI-TOF MS can be applied in patients with PJI by culturing synovial fluid in blood culture bottles for rapid diagnosis.  **Purpose**  This project will be a 2-year project. In the first year, 25 patients with PJI will be enrolled. In the second year, another 25 patients will be recruited. The results of those patients with PJI after reimplantation will be followed. The MALDI-TOF MS will be specifically validated in PJIs in the treatment of debridement only or debridement with implant removal. We predict the bacterial detection time will be accelerated with the aid of MSLDI-TOF MS method. |
| --- |

研究計畫之背景及目的：

請詳述本研究計畫之背景、目的、重要性以及國內外有關本計畫之研究情況，重要參考文獻等。本計畫如為整合型計畫之子計畫，請就以上各點分別述明與其他子計畫之相關性。

| **研究計畫之背景**  人工關節感染是人工關節置換最嚴重的併發症之一 [1]。一旦發生人工關節感染，最重要的是要能正確及快速地找到致病菌，才能得到有效的治療。傳統細菌培養及敏感藥物測試常常會有偽陰性的結果產生，特別是當人工關節表面形成生物膜時。同時，關節液或是人工關節旁的組織檢體必須至少培養14天以上，才能有較正確的結果產生[2]。 使用血液培養瓶來診斷人工關節感染的致病菌已行之多年。它已被證實能有效改善診斷的準確性及特異性 [2]。但使用血液培養瓶仍然耗時，一般平均需21~23小時才能培養出結果[2]。    近期基質輔助雷射脫附離子化質譜儀的出現，已經被採用當作是快速從血液培養瓶中培養出致病菌的工具。使用這個方法時，只要幾滴的血液在自陽性血液培養瓶取出執行此檢驗方法不到1個小時即可檢查出病原菌[2]。而且基質輔助雷射脫附離子化質譜儀只需少量的細菌即可診斷。在文獻上，當使用基質輔助雷射脫附離子化質譜儀診斷尿液培養的周轉時間，已大幅從24小時降到4~6個小時即可檢查出細菌[2]。但基質輔助雷射脫附離子化質譜儀在人工關節感染的應用上，目前仍是個未知數。  **研究計畫之目的**  此這個二年期的研究計劃，在第一年及第二年分別收入25個人工關節感染的病人。在病人接受清創手術或拔除人工關節手術時收集關節液的檢體。目的是檢測基質輔助雷射脫附離子化質譜儀是否能使用人工關節感染的關節液檢體來快速診斷造成人工關節感染的致病菌，提供臨床決策之重要資訊。  **重要參考文獻**  1. Peersman G, Laskin R, Davis J, Peterson M. Infection in total knee replacement: a retrospective review of 6489 total knee replacements. Clin Orthop Relat Res. 2001(392):15-23.  2. Fink B, Makowiak C, Fuerst M, Berger I, Schafer P, Frommelt L. The value of synovial biopsy, joint aspiration and C-reactive protein in the diagnosis of late peri-prosthetic infection of total knee replacements. J Bone Joint Surg Br. 2008;90(7):874-8.  3. Geller JA, MacCallum KP, Murtaugh TS, Patrick DA, Jr., Liabaud B, Jonna VK. Prospective Comparison of Blood Culture Bottles and Conventional Swabs for Microbial Identification of Suspected Periprosthetic Joint Infection. J Arthroplasty. 2016;31(8):1779-83.  4. Peel TN, Dylla BL, Hughes JG, Lynch DT, Greenwood-Quaintance KE, Cheng AC et al. Improved Diagnosis of Prosthetic Joint Infection by Culturing Periprosthetic Tissue Specimens in Blood Culture Bottles. MBio. 2016;7(1):e01776-15.  5. Croxatto A, Prod'hom G, Greub G. Applications of MALDI-TOF mass spectrometry in clinical diagnostic microbiology. FEMS Microbiol Rev. 2012;36(2):380-407.  6. Haiko J, Savolainen LE, Hilla R, Patari-Sampo A. Identification of urinary tract pathogens after 3-hours urine culture by MALDI-TOF mass spectrometry. J Microbiol Methods. 2016;129:81-4. |
| --- |

八、研究方法及進行步驟及執行進度：

1.請細述本計畫採用之研究方法與原因。

2.預計可能遭遇之困難及解決途徑。

3.重要儀器之配合使用情形。

4. 一年期以上之計畫，請分年列述。

5. 如為整合型計畫，請就以上各點分別說明與其他子計畫之相關性。

| **Material and Methods**  **1. Patients and samples**  Patients who have high probability of infection based on the Musculoskeletal Infection Society (MSIS) criteria and are scheduled for debridement only or debridement with implant removal will be invited to enroll the study after signed informed consent.  The synovial joint fluid will be sampled before the arthrotomy at the operation room. Aspirates will be collected under an aseptic technique with an 18-Fr sterile syringe with a minimum amount of 14 cc. The sample will be divided between MALDI-TOF mass spectrometry in standard BCBs (10 cc), wound culture tube (2 cc), and synovial fluid analysis (2 cc). The samples will be delivered to microbiology laboratory within a 2-hour period.  **2. Bacterial culture and conventional identification**  Bacterial identification will be performed by the conventional method using the Vitek 2 system. For the conventional culture, 1 µL of well-mixed synovial joint fluid will be inoculated and spread onto blood agar plates and MacConkey agar plates using a sterile plastic disposable loop. Plates will be incubated in an aerobic atmosphere at 37℃ for 18-24 hr. When bacterial growth is observed, the colonies on blood agar will be counted, and colonies from both types of plates will be identified by using the Vitek 2 system.  **3. MALDI-TOF MS identification**  The suspension obtained following the above sample preparation will be centrifuged at 13,000g for 2 minutes, and the supernatant will be discarded. The pellet will be centrifuged at 13,000g for another 2 minutes prior to the removal of the residual ethanol. Fifty microliters of formic acid (70% v/v) and 50 mL of 100% acetonitrile will be added to the pellet, and mixed thoroughly after each reagent is added. The suspension will be centrifuged again at 13,000g for another 2 minutes, and 1 mL of the supernatant will be spotted onto the steel target plate. Analysis will be performed following air-drying of 1 mL a-cyano-4-hydroxycinnamic acid matrix solution placed onto the dried sample spot in duplicate.  Mass spectra profiles will be acquired using a microflex LT MALDI-TOF mass spectrometer (Bruker Daltonics, Bremen, Germany) following the manufacturer’s settings. Spectra will be recorded in the linear positive mode at a laser frequency of 60 Hz within a mass range from 2000 Da to 20,000 Da. All bacteria identifications will be performed by MALDI-TOF Biotyper RTC and the Bruker MALDI Biotyper 3.1 software and library (4613 isolates; Bruker Daltonics). Criteria used for microorganism analysis and identification will be as recommended by the manufacturer.  **4. Statistical analysis**  Time to identification will be determined as the time from colony formation to the time at which the final result is reported to a physician. Statistical analysis will be performed to compare the three methods using Chi-square tests. The level of statistical significance will be set at p < 0.05.  **預計可能遭遇之困難及解決途徑**  1. 確定感染的個案目前以細菌培養為主要參考標準，然而細菌培養的偽陰性可高達25-40％，因此對於確診感染的條件無法使用細菌培養的結果作為標準。解決方法為使用診斷感染的標準(MSIS criteria)來作為確診的依據。  2. 試驗人數可能不夠。人工關節感染，以高雄長庚的經驗，大概每年20~25個。故此我們延長計劃為2年，以期收到足夠的病人數  **試驗設計**  第一年預計收案25 例人工關節感染案例以進行研究。檢體送檢分成  （1）傳統細菌培養  （2）血液培養瓶  （3）基質輔助雷射脫附離子化質譜儀  第二年預計再收案25 例人工關節感染案例，檢體送檢同樣分成  （1）傳統細菌培養  （2）血液培養瓶  （3）基質輔助雷射脫附離子化質譜儀。  所有病人資料以編號識別保密。如果將來發表研究成果時,受試者的身份仍將保密，並承諾絕不違反受試者的身份之機密性。 研究目的  1. 使用基質輔助雷射脫附離子化質譜儀來診斷人工關節感染的關節液檢體檢測出致病菌所需的時間。  2. 並追蹤感染後接受清創病人的結果或階段性再次置換人工關節病人的結果。 |
| --- |

Chang Gung Medical Research Application

**Rapid diagnosis of prosthetic joint infection by matrix-assisted laser desorption ionization time-of-flight mass spectrometry**

**Background**

Prosthetic joint infection (PJI) remains one of the most catastrophic complications after total joint replacement. To achieve the efficacy of treatment, the causing microorganism must be identified accurately and rapidly [1].

Conventional culture and sensitivity test on agar plates often have falsely negative results as biofilm are formed on the prosthesis. Meanwhile, the synovial fluid or periprosthetic tissue specimens must be incubated for at least 14 days [2]. Blood culture bottles (BCBs) have been used to identify the pathogens in PJI with the improvement of sensitivity and specificity. But BCBs still take considerable time, within a median of 21 to 23 hours, to yield positive results [3,4].

Recently, matrix-assisted laser desorption ionization time-of-flight mass spectrometry (MALDI-TOF MS) has been adopted as a rapid tool to identify bacteria from positive blood culture bottles. In this method, only a few drops of blood form positive blood bottle and subjected to MALDI-TOF MS for a short time (less than one hour) with a small amount of an organism [5]. The turnaround time for identification of urine culture was improved from 24 hours to 4-6 hours when using MSLDI-TOF MS compared with conventional cultures [6]. But it is unknown if MSLDI-TOF MS can be applied in patients with PJI by culturing synovial fluid in blood culture bottles for rapid diagnosis.

**Purpose**

This project will be a 2-year project. In the first year, 25 patients with PJI will be enrolled. In the second year, another 25 patients will be recruited. The results of those patients with PJI after reimplantation will be followed. The MALDI-TOF MS will be specifically validated in PJIs in the treatment of debridement only or debridement with implant removal. We predict the bacterial detection time will be accelerated with the aid of MSLDI-TOF MS method.

**Material and Methods**

**1. Patients and samples**

Patients who have high probability of infection based on the Musculoskeletal Infection Society (MSIS) criteria and are scheduled for debridement only or debridement with implant removal will be invited to enroll the study after signed informed consent.

The synovial joint fluid will be sampled before the arthrotomy at the operation room. Aspirates will be collected under an aseptic technique with an 18-Fr sterile syringe with a minimum amount of 14 cc. The sample will be divided between MALDI-TOF mass spectrometry in standard BCBs (10 cc), wound culture tube (2 cc), and synovial fluid analysis (2 cc). The samples will be delivered to microbiology laboratory within a 2-hour period.

**2. Bacterial culture and conventional identification**

Bacterial identification will be performed by the conventional method using the Vitek 2 system. For the conventional culture, 1 µL of well-mixed synovial joint fluid will be inoculated and spread onto blood agar plates and MacConkey agar plates using a sterile plastic disposable loop. Plates will be incubated in an aerobic atmosphere at 37℃ for 18-24 hr. When bacterial growth is observed, the colonies on blood agar will be counted, and colonies from both types of plates will be identified by using the Vitek 2 system.

**3. MALDI-TOF MS identification**

The suspension obtained following the above sample preparation will be centrifuged at 13,000g for 2 minutes, and the supernatant will be discarded. The pellet will be centrifuged at 13,000g for another 2 minutes prior to the removal of the residual ethanol. Fifty microliters of formic acid (70% v/v) and 50 mL of 100% acetonitrile will be added to the pellet, and mixed thoroughly after each reagent is added. The suspension will be centrifuged again at 13,000g for another 2 minutes, and 1 mL of the supernatant will be spotted onto the steel target plate. Analysis will be performed following air-drying of 1 mL a-cyano-4-hydroxycinnamic acid matrix solution placed onto the dried sample spot in duplicate.

Mass spectra profiles will be acquired using a microflex LT MALDI-TOF mass spectrometer (Bruker Daltonics, Bremen, Germany) following the manufacturer’s settings. Spectra will be recorded in the linear positive mode at a laser frequency of 60 Hz within a mass range from 2000 Da to 20,000 Da. All bacteria identifications will be performed by MALDI-TOF Biotyper RTC and the Bruker MALDI Biotyper 3.1 software and library (4613 isolates; Bruker Daltonics). Criteria used for microorganism analysis and identification will be as recommended by the manufacturer.

**4. Statistical analysis**

Time to identification will be determined as the time from colony formation to the time at which the final result is reported to a physician. Statistical analysis will be performed to compare the three methods using Chi-square tests. The level of statistical significance will be set at p < 0.05.

**Reference**

1. Peersman G, Laskin R, Davis J, Peterson M. Infection in total knee replacement: a retrospective review of 6489 total knee replacements. Clin Orthop Relat Res. 2001(392):15-23.

2. Fink B, Makowiak C, Fuerst M, Berger I, Schafer P, Frommelt L. The value of synovial biopsy, joint aspiration and C-reactive protein in the diagnosis of late peri-prosthetic infection of total knee replacements. J Bone Joint Surg Br. 2008;90(7):874-8.

3. Geller JA, MacCallum KP, Murtaugh TS, Patrick DA, Jr., Liabaud B, Jonna VK. Prospective Comparison of Blood Culture Bottles and Conventional Swabs for Microbial Identification of Suspected Periprosthetic Joint Infection. J Arthroplasty. 2016;31(8):1779-83.

4. Peel TN, Dylla BL, Hughes JG, Lynch DT, Greenwood-Quaintance KE, Cheng AC et al. Improved Diagnosis of Prosthetic Joint Infection by Culturing Periprosthetic Tissue Specimens in Blood Culture Bottles. MBio. 2016;7(1):e01776-15.

5. Croxatto A, Prod'hom G, Greub G. Applications of MALDI-TOF mass spectrometry in clinical diagnostic microbiology. FEMS Microbiol Rev. 2012;36(2):380-407.

6. Haiko J, Savolainen LE, Hilla R, Patari-Sampo A. Identification of urinary tract pathogens after 3-hours urine culture by MALDI-TOF mass spectrometry. J Microbiol Methods. 2016;129:81-4.
