# Supplementary material for: Rapid diagnosis of periprosthetic joint infection from synovial fluid in blood culture bottles by direct matrix-assisted laser desorption ionization time-of-flight mass spectrometry
Source: PLoS One. 2020 Sep 24;15(9):e0239290. doi: 10.1371/journal.pone.0239290 (PMC7515592; doi:10.1371/journal.pone.0239290)
Supplement: Attachment — Submitted filename: Response to Reviewers.docx [file pone.0239290.s004.docx]

**Response to Reviewers**

Re: **Rapid diagnosis of periprosthetic joint infection from synovial fluid in blood culture bottles by direct matrix-assisted laser desorption ionization time-of-flight mass spectrometry**

Dear Editors,

Thank you for the comprehensive review of our manuscript. We appreciate the opportunity to submit a revised version and found the reviewers' comments to be very insightful and contribute considerably to the improvement of our manuscript.

Below we provide our point-by-point responses to all of the reviewers’ comments. We also send this manuscript for English editing. We hope you will find our revised manuscript suitable for publication in *PLOS ONE*.

Sincerely,

Chen-Hsiang Lee

**Reviewer#1**

1. **Line 143. “80 SF samples were taken from 77 patients...” In other words, some samples were taken from the same patients and were correlated. However, the analysis doesn’t discuss how the correlated data were accounted.**
   Ans: Thank you for this and all of your comments. We have rewritten this in page 13, line 158-159: “Two patients had recurrent infections after a two-stage exchange protocol. One patient had bilateral staged knee PJIs at different time points during the study period. Therefore, 80 SF samples were taken and analyzed from 77 patients who fulfilled the MSIS criteria (Table 1).”
2. **Line 195. “All pairwise were significant differences .....”. Please report ANOVA results first. Also, what multiple testing procedure was applied?**
   Ans: We used the Bonferroni post hoc test for multiple comparisons between groups. We have added this to page 12, line 149-151: “In order to fully understand group differences, the Bonferroni post hoc test was used for multiple comparisons when the Kruskal-Wallis test was significant.”

We reported the overall results and group differences on page 20, line 211-226: “The overall difference between the three groups was significant (p < 0.0001). Significant Bonferroni-adjusted post hoc interactions are shown in Fig 2. In pairwise comparisons, there were significant differences in the time of microorganism identification between direct MALDI-TOF MS and routine MALDI-TOF MS (p < 0.0001) or swab culture (p < 0.0001). There was no significant difference between routine MALDI-TOF MS and swab culture (p = 0.0268).”

**Reviewer #2**

1. **The numbers of the patients included in the study is not big enough, there is still needed for the more samples to further verify the conclusion.**
   Ans: Thank you for this and all of your comments. We have added this to address the power analysis on page 12, line 152-154: “Post hoc power analysis showed that a sample size of 80 was sufficient to achieve 89% power to detect an effect size of 0.23 with a significance level of 0.05 using a 2-sided one-way ANOVA test (G Power v3.1, Baden-Württemberg, Germany).”
   We have also added this in the conclusion on page 26, line 287-288: “Further large-scale studies are required to verify the clinical impact and the value of this testing method.”
2. **The presentation of the study is unclear and lacks rigor.**
   Ans: We calculated group differences using the Bonferroni post hoc test for multiple comparisons (page 12, line 149-151): “In order to fully understand group differences, the Bonferroni post hoc test was used for multiple comparisons when the Kruskal-Wallis test was significant.”

We reported the overall results and group differences on page 20, line 211-216: “The overall difference between the three groups was significant (p < 0.0001). Significant Bonferroni-adjusted post hoc interactions are shown in Fig 2. In pairwise comparisons, there were significant differences in the time of microorganism identification between direct MALDI-TOF MS and routine MALDI-TOF MS (p < 0.0001) or swab culture (p < 0.0001). There was no significant difference between routine MALDI-TOF MS and swab culture (p = 0.0268).”

3. **The authors argue that the purpose of the study was to evaluate whether direct MALDI-TOF MS can shorten the time to preliminary strain identification as compare with routine methods (Page 4, Line 61-62). Direct MALDI-TOF MS of course can shorten the time to preliminary strain identification, as subculture is not needed. Thus the argument is meaningless.**
   Ans: Thank you for your valuable opinion. We have rewritten this part of the introduction on page 6, line 52-59: “Theoretically, direct MALDI-TOF MS can decrease the time to preliminary strain identification, as a subculture is not required in this process. This rapid microbial identification method has been used in many infection fields, such as bloodstream infections [8,11,12] meningitis [13,14] and urinary traction infections [15,16]. This method has been shown to have high accuracy for Gram-negative organisms but relatively low accuracy for Gram-positive bacteria [17]. However, Gram-positive bacteria are the most common organisms that cause PJIs [18,19]. However, whether the direct identification method can be efficiently applied in patients with PJIs by culturing SF in BCBs for a rapid and accurate diagnosis has yet to be elucidated.”
4. **The Material and Methods section has several mistakes and detailed information is lacked. Page 6, line 116, where is the ethanol come from?**

Ans: We have rewritten the section of Material and Methods (line 89 on page 8 to line 143 on page 11) and changed a reference [Yonetani S, Ohnishi H, Ohkusu K, Matsumoto T, Watanabe T. Direct identification of microorganisms from positive blood cultures by MALDI-TOF MS using an in-house saponin method. Int J Infect Dis. 2016;52: 37–42. doi:10.1016/j.ijid.2016.09.014].

5. **Line 116-120, to my knowledge, it should be “μL” instead of “mL”, etc.....**
   Ans: Thank you. The unit has been changed to μL.
6. **The results section is badly structure and the description is confusing, major improvement is needed.**
   Ans: The results section describes direct versus routine MALDI-TOF MS, followed by direct MALDI-TOF MS versus swabs, then routine swab culture versus swab.

We have also added the group difference in the time to microorganism identification on page 20, line 211-216: “The overall difference between the three groups was significant (p < 0.0001). Significant Bonferroni-adjusted post hoc interactions are shown in Fig 2. In pairwise comparisons, there were significant differences in the time of microorganism identification between direct MALDI-TOF MS and routine MALDI-TOF MS (p < 0.0001) or swab culture (p < 0.0001). There was no significant difference between routine MALDI-TOF MS and swab culture (p = 0.0268).”

We also added a paragraph in the discussion section to describe our finding in page 22 and 23, line 232-244 “Interestingly, direct MALDI-TOF MS identified 85.3% of Gram-positive organisms and 92.3% of Gram-negative organisms in SF samples in BCBs. Our results are consistent with previous studies which showed that direct MALDI-TOF MS had high accuracy for Gram-negative bacteria but moderate accuracy for Gram-positive organisms [17,22,23]. Zhou et al. demonstrated a higher accuracy for Gram-negative bacteria than for Gram-positive bacteria (92.8 vs. 82.4%) in their in-house protocol for direct MALDI-TOF MS in positive BCBs [8]. In addition, French et al reported an identification rate of 53% for Gram-positive organisms and 89% for Gram-negative organisms [12]. A recent systemic review and meta-analysis of 32 studies reported an overall correct identification rate of 72% for Gram-positive bacteria compared to 92% for Gram-negative bacteria with direct bacterial identification from positive BCBs using MALDI-TOF MS [24]. A possible reason for the lower identification rate of Gram-positive bacteria with MALDI-TOF MS analysis is because Gram-positive bacteria require pre-processing steps due to their more robust cell wall, which decreases the protein extraction rate [25].”
