# Supplementary material for: Rapid diagnosis of periprosthetic joint infection from synovial fluid in blood culture bottles by direct matrix-assisted laser desorption ionization time-of-flight mass spectrometry
Source: PLoS One. 2020 Sep 24;15(9):e0239290. doi: 10.1371/journal.pone.0239290 (PMC7515592; doi:10.1371/journal.pone.0239290)
Supplement: Attachment — Submitted filename: Response to Reviewers.doc [file pone.0239290.s005.doc]

**Response to Reviewers**

Re: **Rapid diagnosis of periprosthetic joint infection from synovial fluid in blood culture bottles by direct matrix-assisted laser desorption ionization time-of-flight mass spectrometry**

Dear Editors,

Thank you for the comprehensive review of our manuscript. We appreciate the opportunity to submit a revised version and found the reviewers' comments to be very insightful and contributed considerably to improving the manuscript.

Below we provide our point-by-point responses to the reviewers’ comments. We also sent the manuscript for English editing. We hope you will find our revised manuscript suitable for publication in *PLOS ONE*.

Sincerely,

Chen-Hsiang Lee MD, MSc

**Editor comment**

**The manuscript was improved, however it requires again a revision. In particular, a language and editing revsion is needed especially for the sentences newly added. Furthermore, the Authors should be more cautious affirming that a subculturing is not needed when the direct identification by MALDI-TOF MS is performed.**

Ans: Thank you for this and all of your comments. The revised edition has been edited by an English native speaker to improve the language writing.

Thanks for your reminder. The most time-intensive process using routine MALDI-TOF MS is the time required for the subculture of specimens before identification. Theoretically, direct MALDI-TOF MS can decrease the time to preliminary strain identification. We have clarified the issue of performing a subculture in the revised version of the manuscript.
